# Can Energy Cost During Low-Intensity Resistance Exercise be Predicted by the OMNI-RES Scale?

**DOI:** 10.2478/v10078-011-0062-5

**Published:** 2011-10-04

**Authors:** Jefferson M. Vianna, Victor M. Reis, Francisco Saavedra, Vinicius Damasceno, Sérgio G. Silva, Fredric Goss

**Affiliations:** 1 Laboratory of motor evaluation of Federal University of Juiz de Fora, Brazil; 2 Department of Sport Sciences, Exercise and Health, University of Trás-os-Montes and Alto Douro (UTAD), Vila Real, Portugal; 3 Research Centre for Sports Sciences, Health & Human Development (CIDESD), Vila Real, Portugal; 4 University Salgado de Oliveira, City Juiz de Fora, Brazil; 5 Federal University of Paraná, Curitiba, Brazil; 6 University of Pittsburgh, Pittsburgh, USA

**Keywords:** resistance exercise, oxygen uptake, rate of perceived exertion

## Abstract

The aim of the present study was to assess the precision of the OMNI-RES scale to predict energy cost (EC) at low intensity in four resistance exercises (RE). 17 male recreational body builders (age = 26.6 ± 4.9 years; height = 177.7 ± 0.1 cm; body weight = 79.0 ± 11.1 kg and percent body fat = 10.5 ± 4.6%) served as subjects. Initially tests to determine 1RM for four resistance exercises (bench press, half squat, lat pull down and triceps extension) were administered. Subjects also performed resistance exercise at 12, 16, 20, and 24% of 1RM at a rate of 40 bpm until volitional exhaustion. Oxygen uptake (VO_2_) and rate of perceived exertion (RPE) using the OMNI-RES were obtained during and after all RE. EC was calculated using VO_2_ and the caloric values of VO2 for non-protein RER. Regression analyses were performed for every RE, using EC as the dependent and RPE as the predictor variable. The triceps extension, lat pull down and bench press, RPE correlated strongly with EC (R > 0.97) and predicted EC with a error of less than 0.2 kcal.min^−1^. In conclusion, RPE using the OMNI-RES scale can be considered as an accurate indicator of EC in the bench press, lat pull down and triceps extension performed by recreational bodybuilders, provided lower intensities are used (up to 24% of 1-RM) and provided each set of exercise is performed for the maximal sustainable duration. It would be interesting in future studies to consider having the subjects exercise at low intensities for longer durations than those in the present study.

## Introduction

The Borg 15 category scale and the Borg CR-10 scale have been employed to rate exertional perceptions in resistance exercise paradigms. However, both of these instruments were developed for use during aerobic exercise and their functional utility was predicated, in part, by on the relation between exertional perceptions and physiological variables such as heart rate and pulmonary ventilation. In an effort to overcome potential limitations of these exertional scaling metrics the OMNI Resistance Exercise Scale (OMNI-RES) was developed specifically for use during resistance exercise. The OMNI-RES employs a unique pictorial format positioned along a fairly narrow numerical response range of 0–10. Verbal descriptors of exertion coincide with numerical categories and mode specific pictures.

The first validation investigation of the OMNI-RES was conducted on a cohort of adult recreationally active weight trainers by Robertson et al. (2003). Subjects performed 3 sets (4, 8 and 12 repetitions) of biceps curl and knee extension exercise at 65% of 1 RM in a counterbalanced order. Each set consisted of 4, 8 and 12 repetitions, presented in a counterbalanced order. Correlations between weight lifted and RPE ranged from 0,79 to 0,91. These findings provided concurrent validation of the OMNI-RES scale for measuring RPE for the active muscle and for the overall body in young recreationally trained male and female weight lifters while performing both upper- and lower-body resistance exercises. However, there is a lack of studies to investigate its applicability in light loads. Moreover, it has been shown the potential of RPE to predict peak VO_2_, in cycle ergometer maximal exercise (Lambrick et al., 2009). Therefore, since low-intensity resistance exercise loads may be used to promote energy cost (EC) during physical activities and since EC may be indirectly measured by steady-state VO_2_, it is interesting to understand how much RPE may serve to quantify EC during RE at those intensities.

Thus, the aim of the present study was to assess the precision of the OMNI-RES scale to predict energy cost (EC) at low intensity in four resistance exercises (RE).

## Materials and Methods

### Subjects

The sample consisted of 17 male recreational bodybuilders who had been training up to 3 times a week for a year (age = 26.6 ± 4.9 years, height = 177.7 ± 0.1 cm, weight = 79.0 ± 11.1 kg and estimated fat mass = 10.5 ± 4.6%), Individuals who used medication which can influence the response to exercise were not included in the sample.

### Protocol

Initially all of the procedures involved in the study were presented to the volunteers, addressing possible risks and benefits. The subjects were instructed to: (a) refrain from exercise 48 h before each experimental trial; (b) refrain from eating for 4 h before each experimental trial; and (c) refrain from the use alcohol, caffeine, or nicotine for at least 24 h before testing. During testing, each subject wore a short-sleeve shirt, shorts, and exercise shoes (Robertson et al., 2003). After signing a declaration of consent to participate in the study, prepared in accordance with the Helsinki Declaration, the subjects completed two questionnaires (PARQ and medical history) to determine their inclusion into the investigation.

During the first experimental session conducted in the morning, height, weight and skin folds were measured and body density and percentage of estimated body fat were calculated. The estimation of fat mass was performed through the sum of skin folds, which were measured by an experienced person with the use of a caliper (Lange, Cambridge Scientific Industries, Cambridge, USA). As such, the following anatomical skin folds were taken: chest; mid-axillary, tricipital, sub scapular, abdominal, supra iliac, and thigh. The anatomical sites were measured according Jackson and Pollock (1978). The formulas used for body density were those proposed by Jackson and Pollock (1978) and Siri (1961) formula was used to convert the body density percent fat.

In this same session the subjects were familiarized with the OMNI-RES. In the second session, held on the same day (the afternoon), the subjects performed a 1RM test in four selected exercises (bench press, half squat, lat pull down. Exercises were performed using standard equipment (Panatta Sport, Italy). The highest load achieved (test and retest) provided the values differed by less than 5% was considered the 1RM (Table 1). Intraclass correlation coefficients ranged between 0.89 and 0.94 in every exercise. Maximal values were 96,7 ± 21,7 kg in the bench press, 126,8 ± 32,3 kg in the half squat, 48,1 ± 9,9 kg in the triceps extension and it was 94,7 ± 13,3 kg in the Lat pull down. A brief description of the exercises follows.

#### Bench press

The subject lay supine on a flat bench with the back fully supported and feet on the ground. Execution was initiated with the elbow in full extension (eccentric phase) a followed the concentric phase of movement, which started with elbows at an angle of 90° flexion.

#### Half squat

The subject stood erect in front of the bar support with the weight supported at shoulder height, with the feet parallel at the same distance away from the shoulders and the hands fixed on the bar. The subject performed a flexion of the lower limbs simultaneously to reach a 90° angle between the thigh and the ground and returned to start position.

#### Triceps Extension

The subject stood with one foot in front of the pulley device and maintained the width of the shoulders; he held the bar with hands in pronation and performed the movement of full extension of the elbow, with subsequent return to starting position.

#### Lat pull down

The subject sat in front of the pulley device with his feet flat on the floor and held the bar with the hands in pronation; he then pulled the bar toward the trunk and when the hands were at the level of his ears he returned the bar to the starting position.

**Figure 1:**
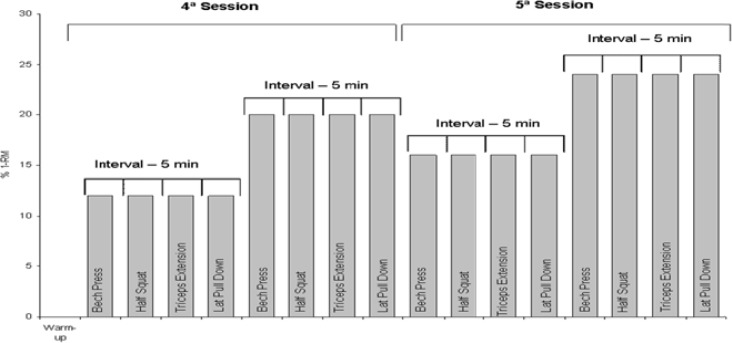
Main experimental sessions

Forty-eight hours later, a fourth session was held to measure the energy cost (EC) during the protocol in the four (4) exercises with two loads. Forty-eight hours later, EC was measured during exercise using two additional loads (fifth session). Load assignment for each session was random. In every exercise, the four loads that were used were 12%, 16%, 20% and 24% of 1-RM. All sessions were performed for each subject at the same time of day (afternoon). Temperature was 20–25 degrees C and relative humidity 35–45%. During the implementation of the protocols with RE, the subjects did not perform any training involving the same muscle groups that were tested. However, they were allowed to perform aerobic training of low intensity and short duration (up to 20min) and other general exercises without loads for different muscle groups (eg, abdominals, stretching). Figure 1 displays the sequence of procedures in the main experimental sessions (4^th^ and 5^th^).

Each exercise at each load was performed until exhaustion, as indicated by the inability to maintain cadence for 2 reps (duration typically ranged between three to five minutes) with intervals of recovery that allowed the VO_2_ to return to resting values. Resting VO_2_ was previously assessed with the subject sitting for 5 minutes. Exercise cadence was controlled by an electronic metronome (Qwick Time TM, China) and the pace of execution was 2 seconds for the eccentric phase and 1 second for the concentric phase (40 rpm = 20 repetitions per minute) (Short and Sedlock, 1997; Haltom et al. 1999). During the execution of all exercises as well as in the first 5min of recovery expired gases were analyzed with an open air circuit (COSMED *® K4b^2^*, Rome, Italy). Throughout the experiment period, the *K4b^2^* unit was calibrated daily according to the manufacturer. VO_2_ was measured breath-by-breath and then smoothed with a 20 s averaging. The EC for each load was calculated from the average VO_2_ in the last minute of exercise and assuming an energy equivalent of 5 cal per ml O_2_, provided a steady-state occurred in VO_2_ (as given by a variation of less than 2 ml.kg^−1^.min^−1^ between consecutive minutes. Immediately after each set of every exercise, the subjects were asked to select a number on the OMNI-RES (Robertson et al., 2003) which represented the exertion of the muscles involved in the task. This measure was recorded immediately after each set in every exercise.

### Statistical analysis

After sphericity assumption was verified with the Mauchly test, a repeated measures analysis of variance was performed to detect the exercise and intensity effects in RPE and its interaction.

Linear regressions were used to investigate the precision of EC prediction as a function of RPE. The standard error of the regression (*S*_y.x_) was used a measure of the goodness of the fit.

Data analysis was performed with the SPSS 16.0 (SPSS Science, Chicago, USA) and the graphics designed with Sigma Plot 10.0 (SPSS Science, Chicago, USA). Data are presented as means and standard deviations. A minimum level of significance of *P* ≤ 0.05 was adopted.

## Results

**Figure 2:**
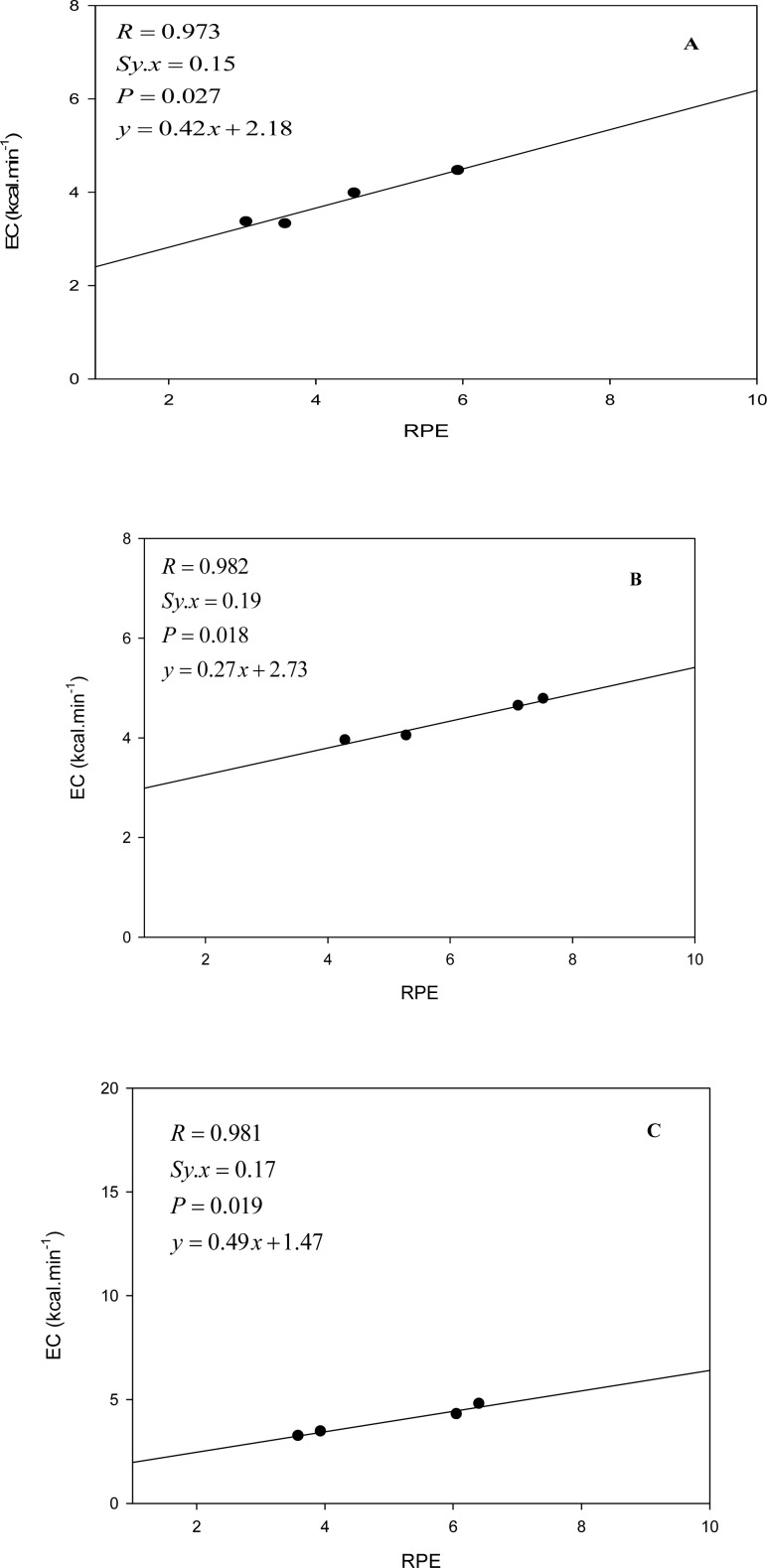
Simple regression analysis between energy cost (EC) and rate of perceived exertion (RPE): Lat Pull down (A), Bench Press (B) and Triceps Extension (C). Regressions were built with mean OMNI-RES and EC values. (N = 17)

The loads that were used in each exercise and the duration of each bout are presented in Table 1. When assessing the variations in RPE (see values also in Table 1) according to the four exercises and to the different loads, a general effect was identified for both independent variables. The RPE increased significantly with the exercise intensity (*P*=0,000; η^2^=0.83) with an exception of the comparison between the first two bouts (12% *vs.* 16%). There were no significant differences between RPE in half squat and in bench press. The RPE during triceps extension was significantly higher compared to every other exercise and the RPE during Lat pull down was significantly lower when compared with every other exercise. Simple linear regressions were established to estimate the EC using RPE (Figure 2).Significant (p< 0,05) regression equations were noted for the bench press, triceps extension and lat pull down. The linear regression that was obtained for the Half squat was not significant

## Discussion

The aim of the present study was to assess the accuracy of equations based on RPE obtained using the OMNI-RES to predict energy cost (EC) during low intensity resistance exercise (RE).The main finding of the present study was that EC can be accurately predicted from RPE during low intensity lat pull down, bench press and triceps extension in recreational body builders. Our results suggest that the accuracy of the prediction model based upon the half squat is not acceptable. Generally, the RPE tended to be higher during triceps extension as compared with the remaining three exercises that were used in the present study. These results suggest that single-joint exercises result higher RPE than multiple joint exercises. This finding is consistent with Lagally et al. (2002^b^) who assessed RPE at intensities of 30 and 90% of 1RM in seven different exercises (both single-joint and multi-joint). Smolander et al. (1998), reported similar differences in RPE in both young and old subjects performing single and multiple joint exercises.

According to Hetzler et al. (1991) RPE is more strongly associated with the accumulation of muscle lactate than with heart rate and blood pressure. This implies that the more localized accumulation of lactate during single-joint movements as compared with multi-joint exercises performed at the same intensity could result in higher RPE (Lagally et al., 2002^a^; Lagally et al. 2002^b^). In addition the present finding that RPE progressively increased with increasing exercise intensity is consistent with the results of multiple investigations. Indeed, Lagally et al. (2004) reported that RPE was greater during resistance exercise performed at 80 than 60% of 1 RM. This observation has also been reported by Lagally and Robertson (2006), Gearhart et al. (2002), Sweet et al. (2004) and Suminski et al. (1997).

Studies also show that RPE increases as a function of the number of repetitions and sets for a given weight (Robertson et al., 2003; Pincivero et al., 2004; Woods et al., 2004). In protocols with eccentric contractions, which used various combinations of loads and repetitions, RPE also increased with the increasing of number of repetitions (Hollander et al., 2003), and this linear increase in line with repetitions increasing (Pincivero et al., 2004). The RPE response during RE is related to the total amount of weight being lifted (i.e. the combination of repetitions and weight). Thus, for sub maximal efforts, the literature confirms that RPE is sensitive to training volume. The current study was not designed to address this issue. However, since our subjects did perform every exercise until exhaustion, the RPE mean values that we have observed must be interpreted as a consequence of the number of repetitions and not only as a response to a specific load. In the future it may be interesting to confirm if the use non-exhaustive low-intensities (below 30% of 1-RM) confirm our findings.

The prescription of aerobic exercise intensity can be based on RPE, as the latter is strongly related to the physiological overload (Robertson, 2001).The present findings suggest that RPE may also have some functional utility as a prescriptive reference to estimate EC during RE. In every exercise, but the half squat, the steady-state criterion was observed. It is true that VO_2_ measures only the aerobic fraction of energy release. It has been suggested that contribution from the anaerobic metabolism in RE may represent up to 39% of the total energy cost (Scott, 2006). However, during low intensities (as in the present study) and as long as the VO_2_ attains a steady-state, the anaerobic fraction may be negligible (due only to the initial O_2_ deficit) and the VO_2_ may represent the overall energy cost.

In the triceps extension, the lat pull down and the bench press, OMNI-RES correlated strongly with EC (*R* > 0.97). In the half squat, the correlation was only moderate (*R* = 0.434) and non-significant (*P* = 0.566). The error in predicting EC would be 0.17 kcal.min^−1^ in the triceps extension, 0.10 kcal.min^−1^ in the bench press and 0.15 kcal.min^−1^ in the lat pull down. This means that in the bench press, for example, our subjects’ EC would be between 4 and 5 kcal.min^−1^ at the exercise intensities that were tested and that using the OMNI-RES their EC could be estimated with a ≈3% error; which is a fairly acceptable precision. In summary, the application of the OMNI-RES can be considered as an accurate predictor of EC in the bench press, lat pull down and triceps extension performed by recreational bodybuilders, provided lower intensities are used (up to 24 % of 1-RM) and provided each set of exercise is performed for the maximal sustainable duration. It would be interesting in future studies to assess the relation between RPE and EC in additional exercises. In addition this line of research could be expanded to include women, the elderly and individuals without previous RE experience.

Using this type of estimation in settings such as schools, gyms, fitness centers, and rehab centers may enable healthcare professionals to prescribe weight loss or weight control programs with greater precision. The possibility to estimate EC from RE based on RPE is therefore highly attractive. Since RE may appear as a series of different combinations of exercises and intensities, further studies are warranted to establish a proper reference that can be used by practitioners.

## Figures and Tables

**Table 1:** Means and standard deviations of load, duration of exercise and rate of perceived exertion at 12, 16, 20 and 24% 1-RM in the four exercises

Exercíse	%1RM	Load (kg)	Duration (sec)	RPE
Bench press	12	11,4±2,6	266,47±29,78	4,29±1,69
	16	15,2±3,3	187,06±29,10	5,35±1,93
	20	18,9±4,3	211,76±37,45	7,12±1,73
	24	22,6±5,0	169,41±33,44	7,59±2,12
Half squat	12	13,9±3,2	264,12±41,39	3,59±2,37
	16	18,8±4,0	192,35±36,83	3,88±2,50
	20	23,6±5,0	215,29±28,53	6,06±2,22
	24	28,3±6,2	181,92±7,26	6,41±2,32
Triceps extension	12	5,5±1,1	255,88±30,22	6,06±1,78
	16	7,4±1,6	178,82±24,97	5,65±1,46
	20	9,4±1,9	206,47±29,78	7,18±1,33
	24	11,2±2,4	183,53±14,55	7,41±1,37
Lat pull down	12	11,2±1,6	264,71±30,44	3,06±1,52
	16	14,8±2,1	187,06±29,10	3,59±2,32
	20	18,5±2,8	222,35±28,18	4,53±2,03
	24	22,2±3,4	183,53±14,55	5,94±2,11
